# Editorial: Extracellular vesicles in cancer research: a new era for therapeutic interventions

**DOI:** 10.3389/fphar.2025.1687011

**Published:** 2025-12-01

**Authors:** Tejveer Singh

**Affiliations:** Translational Oncology Laboratory, Department of Zoology, Hansraj College, University of Delhi, Delhi, India

**Keywords:** nanovesicle, phytochemical, ethnopharmaclogy, cancer, therapy

## Abstract

**Figure:**
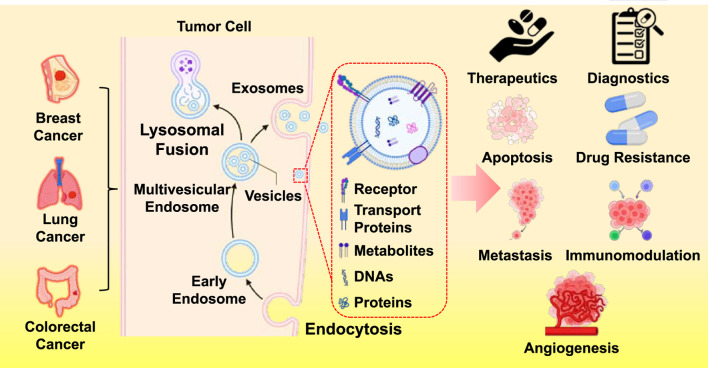

Cancer research has advanced to an exciting point where the majority of the most recent technologies are progressively integrating with conventional knowledge. This convergence has enabled the integration of traditional medicinal knowledge into modern oncology, particularly in cases where modern medicine faces limitations. Recent publications in Frontiers journals, covering topics from exosome-based diagnostics, lung cancer pharmacology, breast cancer immunology, and the ethnomedicine of China’s She minority, vividly demonstrate this shift. Together, these publications highlight the future of oncology, where the tumor microenvironment can be modified using natural bioactive compounds and simultaneously using the body’s own immune and signalling systems.

Li et al., demonstrated cancer is tightly linked to signalling networks, like proliferation driven by tumor-derived extracellular vesicles (EVs) in breast cancer, whereas Wang et al.’s research explored the use of tumor-derived exosomes (TDEs) in cancer diagnostics and treatment (Li et al.; Wang et al.). Extracellular vesicles are recognized as complex messengers that rewire immune cells, alter the vasculature, and initiate metastases. Previously, they were believed to be cellular waste disposal units. In breast cancer, EVs can cause T-cell malfunction, suppress dendritic cell activity, and polarize macrophages toward tumor-supportive phenotypes. They also weaken the impact of targeted therapies by transferring drug-resistant factors, like TGF-β1 and PD-L1, to otherwise sensitive cells. On the other hand, TDE-focused research has highlighted their promise as therapeutic vehicles, delivering medications or vaccinations with exceptional tissue selectivity, and as diagnostic biomarkers, using liquid biopsies to identify cancer-specific molecular cargo. These results suggest a two-pronged strategy: creating vesicles to fight cancer. However, for clinical use, safety profiling, scalable production, and robust reproducibility in isolation methods are mandatory prerequisites.

If EVs serve as the tumor’s secret messaging system, then natural products may represent one of the most flexible and effective defenses. By eradicating the CHRM3/PI3K/AKT and CHRM3/MAPK pathways, natural compounds can disrupt the lung cancer’s ability to proliferate, migrate, and invade. Importantly, this effect endures in xenograft models, suggesting *in vivo* relevance. Acetylcholine activates G-protein-coupled receptors called muscarinic receptors, which are important for both the central and parasympathetic nervous systems. In this instance, the muscarinic receptor CHRM3 acts as a toggle, a cancer-promoting switch (Wu et al.). Controlling the tumor microenvironment, immune modulation, and apoptosis—these are the multi-target, multi-mechanism anticancer effects of medicinal herbs, particularly those rich in flavonoids, like *Melastoma dodecandrum* and *Pimpinella diversifolia,* as per Miao et al.. Notably, some natural products, like homoharringtonine, have already transitioned into clinical use for the treatment of leukemia.

Ethnopharmacological approaches conclusively merit integration into precision oncology frameworks that combine omics-targeted system identification, engineered biobased delivery systems like EVs, and rigorous, validation-dense clinical trial frameworks. This herbal evidence is positioned within a wider abstraction that natural substances have a dominant and holistic effect by acting on multiple nodes within cancer signaling networks, thereby mitigating the risk of single-target resistance. The TDE literature focuses on their remarkable therapeutic multitasking and their role in cancer. Exosomes can be engineered to carry immune stimulants, RNA therapies, or even chemotherapy agents (Li et al.). Additionally, engineered exosomes can serve as “liquid fingerprints” of cancers and outperform many synthetic nanoparticles in immune evasion, biocompatibility, and targeted delivery (Zhang et al.). When combined with adjunct therapeutics, such as photothermal or sonodynamic therapies, they could theoretically eliminate tumors through multiple pathways. However, Wang et al. and Li et al. highlight the persistent safety concerns with TDEs, attributing them to the very endogenous resemblance vesicles that make them so appealing. To mitigate the risks of unintentional immune suppression, off-target actions, or secondary tumor development, engineers need to take safety measures. This paradigm shift in oncology from directly targeting tumor cells to altering the environment in which they reside is reflected in this confluence. The idea has been confirmed by immunotherapies, ranging from CAR-T cells to checkpoint inhibitors. The same immunological axis can now be modulated with new methods thanks to nanovesicle engineering and chemicals produced from plants. Standardization and quality control are two recurring issues that arise because the composition of natural chemicals varies based on harvest, storage, and processing. Strict isolation and characterisation procedures are necessary for EVs. It is difficult to produce clinical-grade exosomes or herbal extracts on a large scale without sacrificing their bioactivity. Although molecular-level effects are still mostly unknown, route mapping (e.g., CHRM3/PI3K/AKT) is progressing (Wu et al.). Ultimately, bridging preclinical promise to clinical utility will require robust mechanistic studies, regulatory frameworks, and comprehensive physician training.

Here, integration offers far more promise than a contest between the old and the new. One could imagine a treatment in which a companion herbal extract strengthens systemic immunity, while an engineered exosome containing a she-derived flavonoid targets metastatic breast cancer cells, alters macrophage phenotype, and delivers a gene-silencing payload to reverse drug resistance. This is the logical conclusion of the tendencies these studies outline; it is not science fiction. However, interdisciplinary cooperation is necessary to get there. Clinical trialists, nanotechnologists, molecular oncologists, and ethnobotanists must converge to translate these synergies into reality. Instead of isolating these disciplines, funding methods ought to incentivize efforts that bridge them.

Cancer research is adopting an unparalleled range of approaches, from the molecular machinery of the tumor cell to the mountains of southeast China. Repurposing nature’s chemical arsenal, disrupting malignant communication networks, and delivering precise treatments with surgical accuracy, thanks to the publications examined here. No one strategy will prevail in the fight against cancer. However, by combining the ancient with the modern—engineered exosomes, polysaccharides, pathway inhibitors, and herbal medicine—we have a greater chance than before. A standardized herbal composition and a nanovesicle infusion, both certified by the same rigorous science and originating from distinct chapters of human invention, may be prescribed in the same sentence by the oncology clinic of the future. This is not merely translational medicine; it is transformational medicine. EV targeting, AMPs, and She medicine chemicals are interwoven through immunomodulation. The tumor microenvironment can be modified toward immune recognition and destruction by reversing T cell exhaustion, halting pro-tumor macrophage polarization, or breaking immunosuppressive cytokine loops.

